# Comparative metabolomics reveals serum metabolites changes in goats during different developmental stages

**DOI:** 10.1038/s41598-024-57803-7

**Published:** 2024-03-27

**Authors:** Qing Li, Tianle Chao, Yanyan Wang, Rong Xuan, Yanfei Guo, Peipei He, Lu Zhang, Jianmin Wang

**Affiliations:** 1 Shandong Provincial Key Laboratory of Animal Biotechnology and Disease Control and Prevention, College of Animal Science and Veterinary Medicine, Shandong Agricultural University, Tai’an, 271018 Shandong China; 2 Key Laboratory of Efficient Utilization of Non-Grain Feed Resources (Co-construction by Ministry and Province), Ministry of Agriculture and Rural Affairs, Shandong Agricultural University, Tai’an, 271018 Shandong China

**Keywords:** Animal breeding, Functional clustering

## Abstract

Goats can provide meat, milk and skins for humans and are livestock with high economic benefits. However, despite their economic significance, the comprehensive analysis of goats’ serum metabolic profile and its intricate alterations throughout their developmental journey remains conspicuously absent. To investigate the stage-specificity and dynamic change characteristics of metabolites during the growth and development of goats, this study compared the alterations in serum hormone levels and serum biochemical markers across different developmental stages of female goats (1, 60, 120 and 180 days old; n = 5). Additionally, a serum untargeted LC–MS metabolomics analysis was conducted. A total of 504 DAMs were identified with age. The results indicated that PE, PC, Lyso-PE, Lyso-PC and FAFHA may play important roles in lipid metabolism in goats after birth. Weighted gene co-expression network analysis (WGCNA) identified two metabolite modules (Turquoise and Yellow) and key metabolites within these modules that were significantly associated with phenotypic features. l-carnitine may be a metabolite related to muscle development in goats. The findings of this study demonstrate notable variations in serum metabolites across distinct developmental phases in goats. Lipids and organic acids play important roles in different developmental stages of goats.

## Introduction

Goats (*Capra hircus*) are significant in the global livestock industry, yielding a diverse range of invaluable products. These include high-protein milk and meat, as well as high-quality sheepskin and cashmere products^1,2^. In contrast to other large ruminants, goats exhibit shorter reproductive cycles, accelerated growth rates, and enhanced adaptability to diverse environments^3,4^.

Metabolomics, through the analysis of alterations in the compositional makeup of metabolites within an organism and their corresponding metabolic pathways, provides a visual and effective means to depict the physiological changes occurring within the organism itself^5–8^. In animal husbandry production, metabolomics has gained significant usage and popularity in recent years. Song et al. utilized serum metabolomics to identify potential serum biomarkers of meat quality related to Shaziling and Yorkshire pigs^9^. Zhang et al. analyzed and characterized the serum metabolome of sheep under overgrazing or light grazing conditions, and identified potential biomarkers associated with the consequences of overgrazing^10^.

Compared to other domestic animals, goat metabolomics is relatively limited. Studies have shown that through the analysis of the serum metabolome of female Guanzhong goats, it was found that there were differences in glucose and lipid metabolism in high and low daily weight gain goats^11^. Metabolomic analysis of Spanish goats affected by diet and stress has shown that fatty acid metabolism is the main energy pathway in all goat populations during prolonged stress^12^. In addition, some studies have shown that lipid and amino acid metabolism pathways in goats before and after calving have undergone significant changes^13^.

The Jining grey goat is an excellent local breed in China, characterised by early sexual maturation and high reproductive capacity. It reaches the initial estrus phase at 2–3 months after birth and achieves sexual maturity at 4 months of age^14^. During the stage of sexual development, significant changes occur in goats’ endocrine and other physiological states. However, the dynamic variations of goat serum metabolites and their potential regulatory mechanisms during the process of sexual development have not been reported yet.

Based on this, the present experiment integrates the biochemical indicators and hormone levels of Jining grey goat serum. The utilization of LC–MS/MS for non-targeted metabolomics analysis was implemented, and the variations in goat serum metabolite levels during four time points of sexual development (1 (D1), 60 (D60), 120 (D120), and 180 (D180) days old) are examined. This aims to comprehend the physiological changes in goat development and lay a solid groundwork for further investigations into the growth and development of goats, while also providing potential valuable biomarkers.

## Materials and methods

### Animal and sample collection

The experiment included a total of 20 female Jining Grey goats, sourced from the Jining Grey goat breeding farm (Jiaxiang County, Shandong Province, China). The goats selected for the study were at the ages of one day (D1, n = 5), 60 days (D60, n = 5), 120 days (D120, n = 5), and 180 days (D180, n = 5)^15^. All the Jining grey goats were not in estrus or in interestrus. Under identical environmental and nutritional conditions, all goats in collective confinement can freely access food and water. At the same time, 10 ml of blood was drawn from the jugular vein of 20 goats. Allow it to stand for one hour and then centrifuge at a speed of 3000*g*/min for 10 min. Transfer the supernatant to a centrifuge tube and store it in liquid nitrogen for subsequent measurement of serum hormones, serum biochemical markers, and metabolomic analysis. (To minimize technical errors, each goat will undergo metabolomic sequencing twice.)

### Serum biochemistry and serum hormone measurements

The serum levels of alanine aminotransferase (ALT), aspartate aminotransferase (AST), alkaline phosphatase (ALP), total protein (TP), albumin (ALB), globulin (GLB), creatine kinase (CK), glucose (GLU), triglycerides (TG), total cholesterol (TC), high-density lipoprotein (HDL), and low-density lipoprotein (LDL) were quantified using the fully automated biochemistry analyzer Hitachi 7020 (Hitachi, Tokyo, Japan). Serum hormone levels, including insulin-like growth factor (IGF-1), progesterone (P), estrogen (E2), growth hormone (GH), and leptin (LEP), were measured by a double-antibody sandwich method using ELISA kit (MDBio, Qingdao, China). The ELISA plates were read using an enzyme immunoassay analyzer (Thermo Scientific, Shanghai, China), and the optical density values were recorded. Standard curves were plotted, and the concentrations of each sample were calculated accordingly.

### Serum metabolite extraction and LC–MS/MS analysis

Transfer 100 µl of goat serum into an EP tube, followed by the addition of 400 µl of an aqueous solution containing 80% methanol, after centrifugation at 4 ℃ for 20 min with a force of 15,000*g*, the subsequent procedure was conducted. Obtain a suitable volume of the supernatant and dilute it with water of mass spectrometry-grade quality until the methanol concentration reaches 53%. Centrifuge the mixture again at 15,000*g* and 4 ℃ for 20 min. The supernatant was collected for analysis using LC–MS after the separation process. Employ the Vanquish UHPLC chromatograph for metabolite separation, setting the column temperature at 40 ℃ and the flow rate at 0.2 ml per minute. Acquire the mass spectrometry signals of the samples in both positive and negative ion scanning modes. For positive mode analysis, employ 0.1% formic acid as mobile phase A, while utilizing methanol as mobile phase B. In the negative mode, opt for methanol as mobile phase B, and employ a mobile phase A comprising a 5 mM ammonium acetate solution adjusted to pH 9.0.

To configure the spray, the ESI source was adjusted to a voltage of 3.5 kilovolts (kV), sheath gas flow rate of 35 pounds per square inch (psi), and auxiliary gas flow rate of 10 l/min. The scanning range was set at *m/z* 100–1500. The ion transfer tube temperature was maintained at 320 °C, while the ion guide RF level was set to 60. The polarity selection was either positive or negative ion mode, and the MS/MS second-level scans utilized data-dependent scanning.

### Metabolite identification and analysis

To process the output data (.raw) files, import them into the Compound Discover software (Version 3.1, Thermo Fishier Scientific). Align peaks from different samples by setting retention time deviation to 0.2 min and mass deviation to 5 ppm, and quantitate peak areas. Subsequently, combine targeted ions and estimate molecular formulas using molecular ion peaks and fragment ions. These results can then be compared with databases such as mzCloud (https://www.mzcloud.org/), mzVault, and Masslist for further analysis. To remove any background ions, utilize blank samples, and normalize the initial quantification outcomes according to the following formula: the quantification value of the raw sample/(sum of quantification values for all metabolites in the sample/total quantification values of metabolites in QC sample), to obtain relative peak areas. Finally, delete compounds with CVs greater than 30% from the QC sample to obtain the identification and relative quantification results of metabolites^16^.

The biological functional annotation of metabolites was conducted utilizing Kyoto Encyclopedia of Genes and Genomes (KEGG; http://www.kegg.com), LIPID MAPS (Lipidmaps; www.lipidmaps.org), and Human Metabolome Database (HMDB; http://www.hmdb.ca). The metaX software (version 1.4.2)^17^ was employed for PCA and PLS-DA analyses, and the variable importance in projection (VIP) was calculated. Differential metabolites were selected based on VIP > 1, *P* < 0.05, FC > 1.5, or FC < 0.67. *P*-value of metabolites between the two groups was evaluated through T-tests. Furthermore, metabolic pathway enrichment analysis of differential metabolites was carried out using the KEGG database. Volcano plots and cluster heatmaps were generated using R software with the ggplot2 and pheatmap packages. while fuzzy c-means clustering analysis was performed using the mfuzz package (version 2.62.0) for time clustering analysis^18^.

### WGCNA analysis

Construct a goat serum differential metabolite co-expression network using WGCNA (V 1.70) through the R package to explore the correlation between differential metabolites and phenotypic indicators such as serum hormones and serum biochemistry ^19^. The parameter settings are as follows: β = 7, CutHeight = 0.8, minSize = 30, and other parameters use default values. Filter hub DAM by using |KME|> 0.5.

### Statistical analysis

Phenotypic indicators such as serum biochemistry and serum hormones were analyzed using SPSS 22.0. The analysis involved performing an ANOVA and employing the LSD method for multiple comparisons. The mean ± standard error of the mean (SEM) was used to present the statistical results, and significance was defined as *P* < 0.05.

### Ethics statement

The study received approval from the Animal Care and Use Committee of the Shandong Agricultural University’s ethics committee under the reference SDAUA-2023-157. Efforts were also made to minimize the discomfort of the animals during the experimental procedures, in accordance with the ARRIVE guidelines (https://arriveguidelines.org). The implementation of all methods adheres to pertinent guidelines and regulations.

## Results

### Analysis of serum biochemical and serum hormone phenotypic indicators

**Figure 1:**
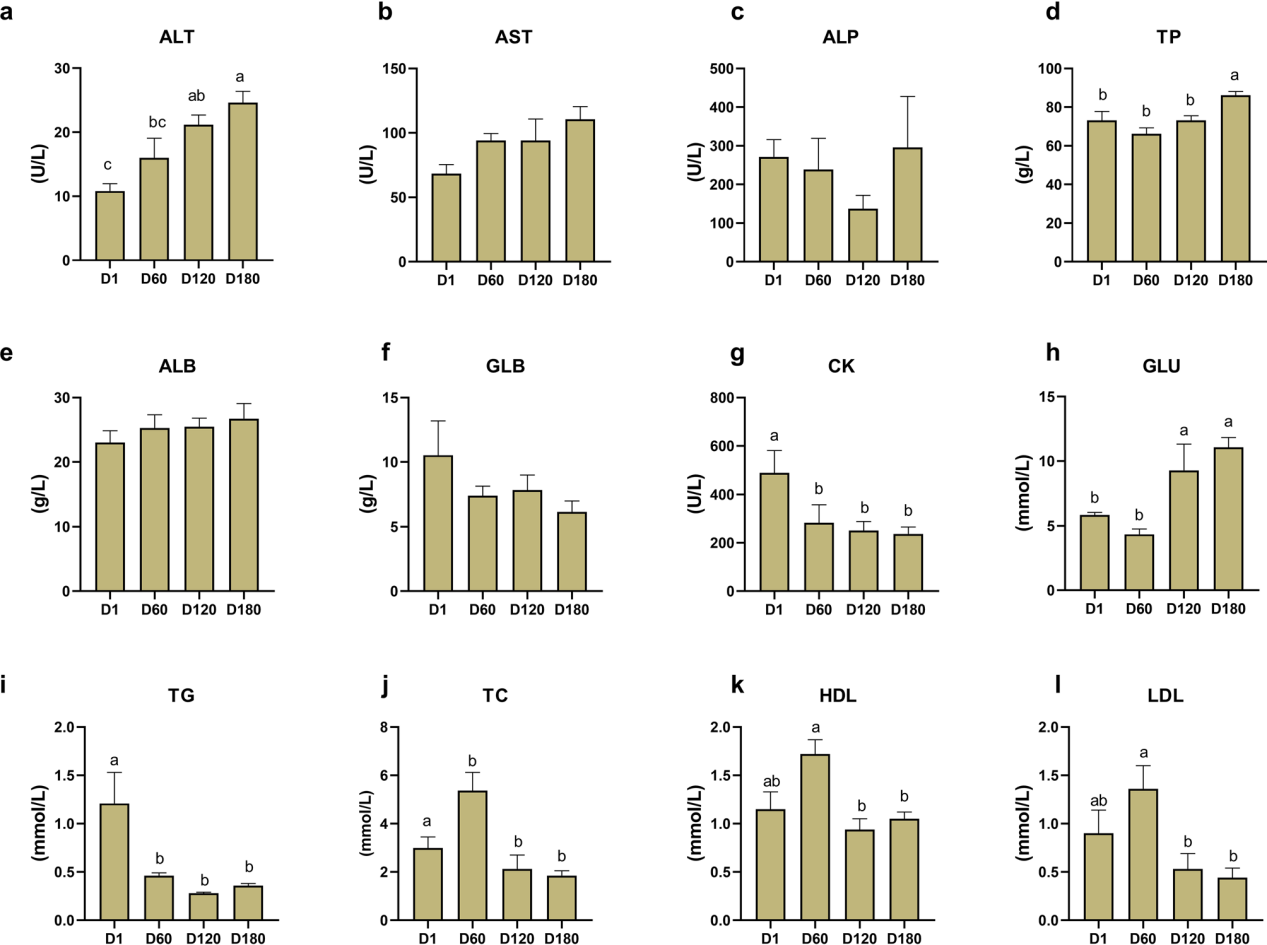
Comparison of serum biochemical indices at different ages (D1, D60, D120, and D180) in the Jining Grey goats. (**a**) Alanine aminotransferase (ALT). (**b**) Aspartate aminotransferase (AST). (**c**) Alkaline phosphatase (ALP). (**d**) Total protein (TP). (**e**) Albumin (ALB). (**f**) Globulin (GLB). (**g**) Creatine kinase (GB). (**h**) Glucose (GLU). (**i**) Triglycerides (TG). (**j**) Total cholesterol (TC). (**k**) High-density lipoprotein (HDL). (**l**) Low-density lipoprotein (LDL). The data are presented as means ± standard error of the mean (SEM) (n = 10). Different lower-case letters indicate significant differences between groups (*P* < 0.05).

**Figure 2:**
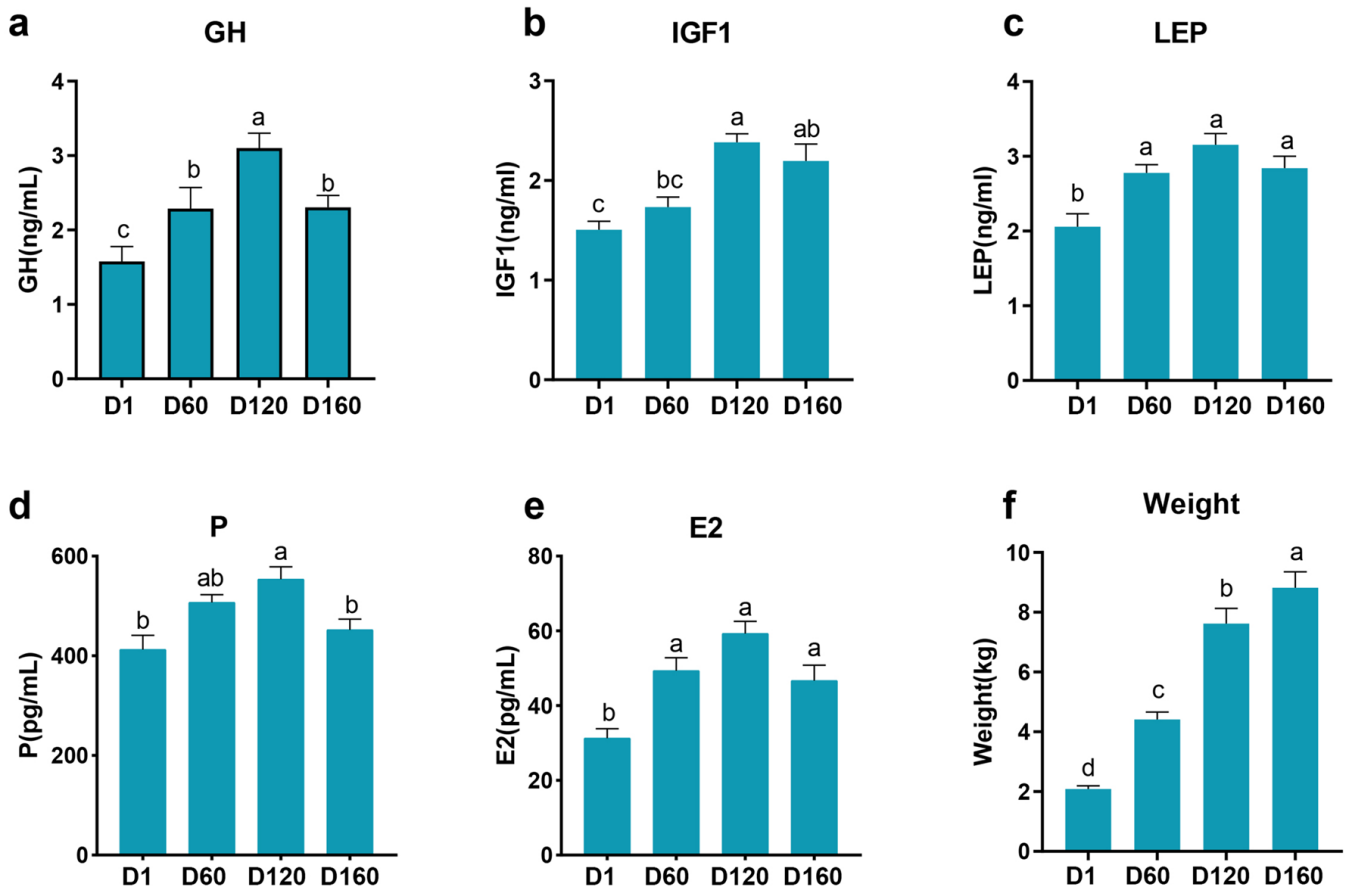
Comparison of weight and serum hormone levels at different ages (D1, D60, D120, and D180) in the Jining grey goats. (**a**) Growth hormone (GH). (**b**) Insulin-Like Growth Factor 1 (IGF1). (**c**) Leptin (LEP). (**d**) Progesterone (P). (**e**) Estrogen (E2). (**f**) Weight. The data are presented as means ± standard error of the mean (SEM) (n = 10). Different lowercase letters indicate significant differences between groups (*P* < 0.05).

This study conducted a biochemical analysis of serum indicators at different ages of Jining grey goats. As shown in Fig. 1, the indicators ALT, TP, CK, GLU, TG, TC, HDL, and LDL of Jining Grey goats exhibited significant differences with increasing age (*P* < 0.05). The ALT level gradually increased with age. The TP and GLU levels showed a trend of initially decreasing and then increasing with age and reached a significant increase in content at D120 to D180 for TP (*P* < 0.05) and at D60 to D120 for GLU (*P* < 0.05). The CK level progressively decreased with age, significantly decreasing during the period from D1 to D60 (*P* < 0.05). The levels of TCHO, HDL, and LDL showed a pattern of initial increase followed by a decrease with age. These content levels reached their peak at D60. Among them, TC content significantly increased from D1 to D60 (*P* < 0.05), and significantly decreased from D60 to D120 (*P* < 0.05), while HDL and LDL content significantly decreased from D60 to D120 (*P* < 0.05). The TG content significantly decreased from D1 to D60 (*P* < 0.05). We also examined serum hormone levels (Fig. 2), which showed that GH was significantly elevated from D1 to D60 (*P* < 0.05). However, there were no notable changes observed between D60 and D120. Subsequently, there was a significant elevation in GH from D120 to D180 (*P* < 0.05). LEP, IGF-1, P, and E2 showed the same trend of change and were significantly elevated from the D1 period to the D60 period (*P* < 0.05).

In addition, the body weight index analysis of goats showed that the weight increased significantly with increasing age (*P* < 0.05), and the growth rate of body weight from D1 to D120 was higher than that from D120 to D180, and the results indicated that the period of D1 to D120 of the Jining Grey goats was a critical stage of growth and development after birth.

### Overview of serum metabolome at different developmental stages

**Figure 3:**
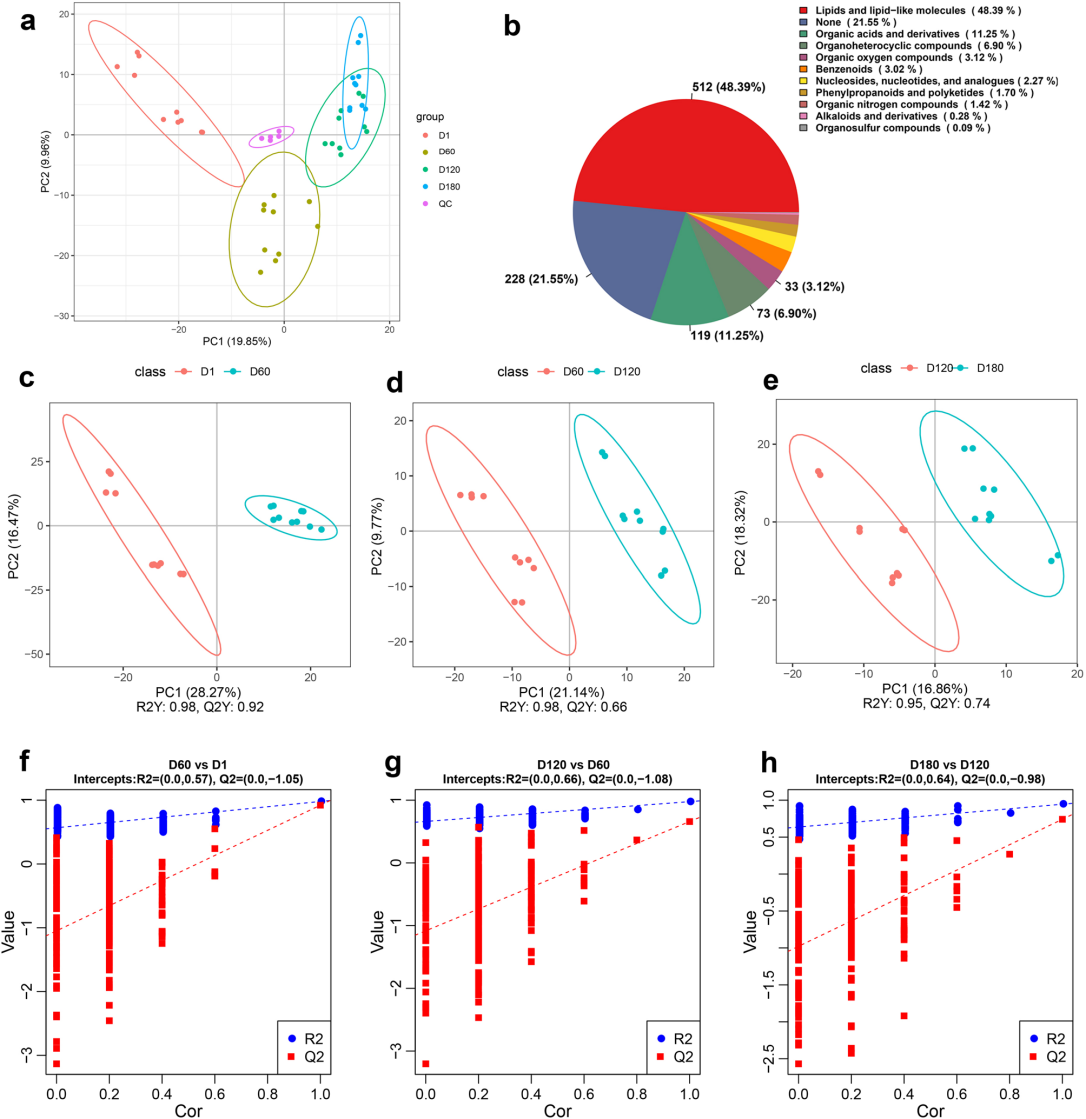
Multivariate statistical analysis of serum metabolomic data based on LS-MS/MS. (**a**) PCA of serum metabolomic profiles in Jining grey goats at different ages. (**b**) Composition analysis of all identified metabolites in serum metabolomic profiles of Jining Grey at different ages. (**c**–**e**) Score plots of PLS-DA for the metabolomic data comparisons: D60 vs. D1, D120 vs. D60, D180 vs. D120. (**f**–**h**) Permutation test results of PLS-DA models for the metabolomic data comparisons: D60 vs. D1, D120 vs. D60, D180 vs. D120.

To investigate the differences in serum metabolism among goats of different ages, this study performed non-targeted metabolomic analysis on serum samples from D1, D60, D120 and D180 Jining grey goats. To obtain real and reliable metabolome data, we first conducted quality control on QC samples, and the results showed that QC samples had good repeatability (Supplementary Table S1, Supplementary Fig. S1). To better examine the differences between groups, this study performed PCA and PLS-DA analyses according to age comparisons. PCA analyses showed that there were variations of differences in metabolites among the D60 vs. D1, D120 vs. D60, and D180 vs. D120 groups (Fig. 3a), suggesting that metabolites changed significantly during the developmental process of these four periods. In total, 1058 metabolites were detected, representing 11 distinct metabolite types (Supplementary Table S2; Fig. 3b). To further test the reliability of the metabolomics data, the metabolite levels in serum were evaluated using the PLS-DA model (Fig. 3c–h), and the parameters of R^2^Y and Q^2^Y were both > 0.5, and the PLS-DA model had high explanatory (R^2^Y) and predictive (Q^2^Y) power, and the Y-axis intercept in the permutation test results was less than 0, which indicated that the model had not been overfitted, and had good predictive ability, and the metabolome data had high reliability.

Based on the analysis of PLS-DA, with VIP > 1.0, *P*-values < 0.05, and fold changes (FC) > 1.5 or FC < 0.667, we screened for differential metabolites in the serum of Jining Grey goats at different ages. In total, 546 differential metabolites were identified.

**Figure 4:**
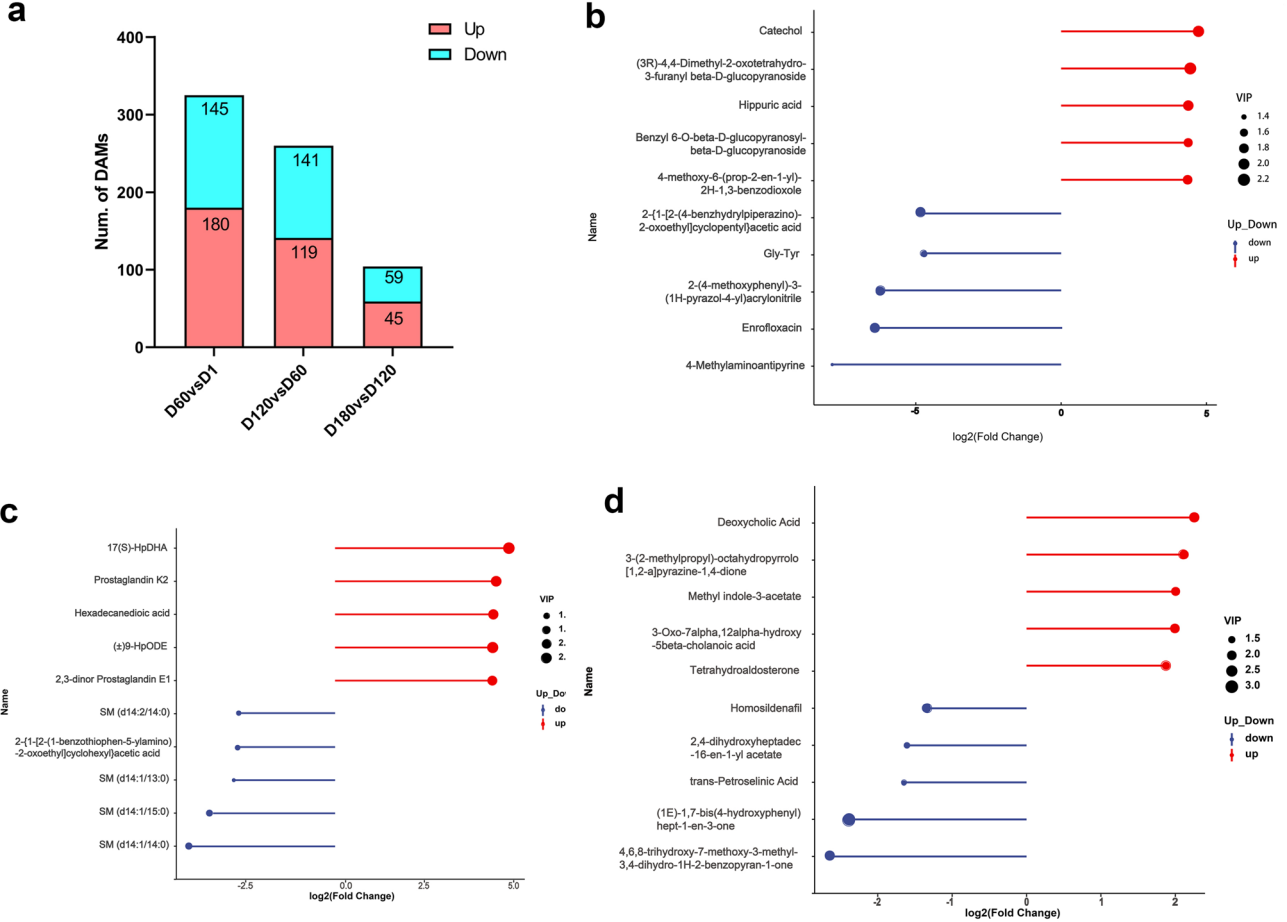
Overview of differential metabolites in serum metabolomics at different developmental stages in Jining Grey goats. (**a**) Histogram of the number of differential metabolites in serum metabolomics. (**b**–**d**) Bar plots of the top 10 metabolites (based on log2FC) between D60 vs. D1, D120 vs. D60, D180 vs. D120.

The results showed that 180 metabolites were up-regulated and 145 metabolites were down-regulated in serum at 60 days of age compared to 1 day of age; 141 metabolites were up-regulated and 119 metabolites were down-regulated in serum at 120 days of age compared to 60 days of age; and 59 metabolites were up-regulated and 45 metabolites were down-regulated in serum at 180 days of age compared to 120 days of age (Fig. 4a, Supplementary Table S3–S5). The highest number of differential metabolites was found between D1 and D60, the lowest between D120 and D180, and the number of DAMs became progressively lower with increasing age. To further understand the overall metabolite dynamics during the development of Jining grey goats, we plotted volcano plots and cluster heatmaps for all detected differential metabolites (Fig. S2), and generated box plots for the top 10 differential metabolites ranked by log2FC (Fig. 4b–d, Supplementary Table S6). Compared with 60 days of age, the contents of SM (d14:1/14:0), SM (d14:1/15:0), SM (d14:1/13:0) and SM (d14:2/14:0) at 120 days of age underwent a significant. Deoxycholic Acid content at 180 days of age was 4.58 times higher than that at 120 days of age.

### Changes in serum fatty acid metabolism at different developmental stages

**Figure 5:**
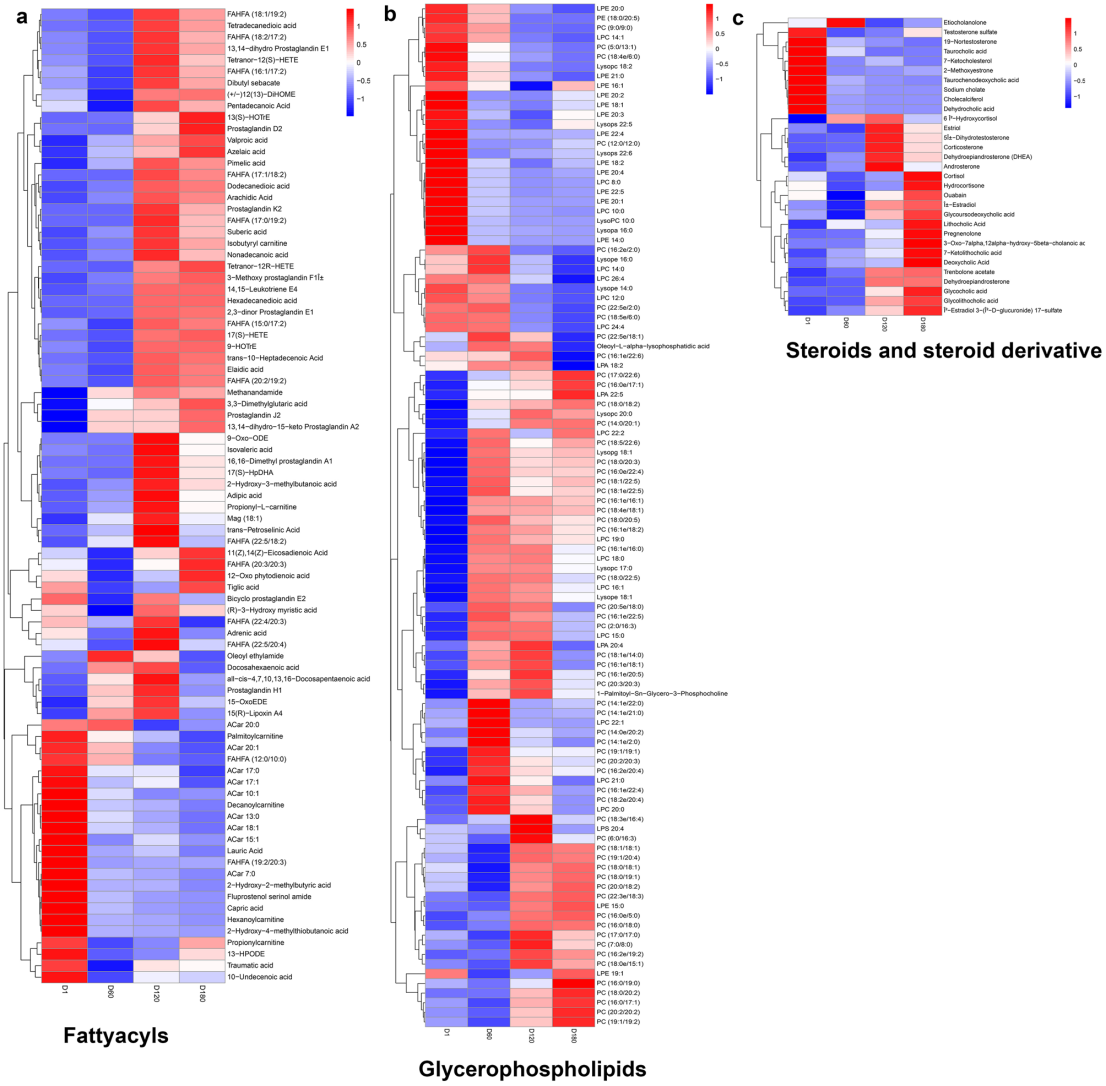
Heatmap of lipid metabolites in the serum of Jining Grey goats at different growth stages. (**a**) Fatty acyls. (**b**) Glycerophospholipids. (**c**) Steroids and steroid derivatives. Metabolites with high expression levels are represented by red squares, while those with low expression levels are indicated by blue squares.

The classification of differential metabolites showed that lipids and lipid-like metabolites were the most abundant metabolites (Supplementary Fig. S3). This study observed that the various classes of metabolites showed different expression patterns, which implies that they play different roles in the development of goats. Fatty acids and lipid compounds were the most abundant metabolite types in the DAMs detected in this study, consisting mainly of glycerophospholipids, fatty acids, steroids and steroid derivatives, and sphingomyelin lipids (Fig. 5a). Metabolites were the highest in variety, and in our study, the glycerophospholipids with altered expression levels consisted mainly of phosphatidylcholine (PC), phosphatidylethanolamine (PE), and (Lyso-PC and Lyso-PE) (Fig. 5b). Among them, PC had the lowest level of expression at postnatal age, which increased gradually with day-old. In fatty acid classification, Acar 20:0, Acar 20:1, Acar 17:0, Acar 17:1, Acar 10:0, Acar 13:0, Acar 18:1, Acar 15:1, and Acar 7:0 were highly expressed at D1 period and that the FAHFA content gradually increased with age, FAHFA (18:1/ 19:2), FAHFA (18:2/17:2), FAHFA (16:1/17:2), FAHFA (17:2/18:2), FAHFA (17:0/19:2), FAHFA (20:2/19:2), FAHFA (22:5/18:2), FAHFA (20:3/20:3), FAHFA (22:5/20:4) were highly expressed during D120 and D180. Steroid hormones exhibit three distinct expression patterns across different developmental stages (Fig. 5c). Among the fatty acids, glycerophospholipid meAt 1 day of age, notable high expressions are observed for Testosterone sulfate, 19-Nortestosterone, Taurocholic acid, 7-Ketocholesterol, Sodium cholate, and other related compounds. Conversely, at 120 days of age, prominent high expressions were found for 5α-dihydrotestosterone, dehydroepiandrosterone (DHEA), androsterone, estriol, α-estradiol, and similar substances. Furthermore, at 180 days of age, significant high expressions were detected for dehydroepiandrosterone, 7-ketolithocholic acid, glycoursodeoxycholic acid, glycocholic acid, cholanoic acid, deoxycholic acid, etc.

### Changes in organic acid metabolism at different developmental stages

**Figure 6:**
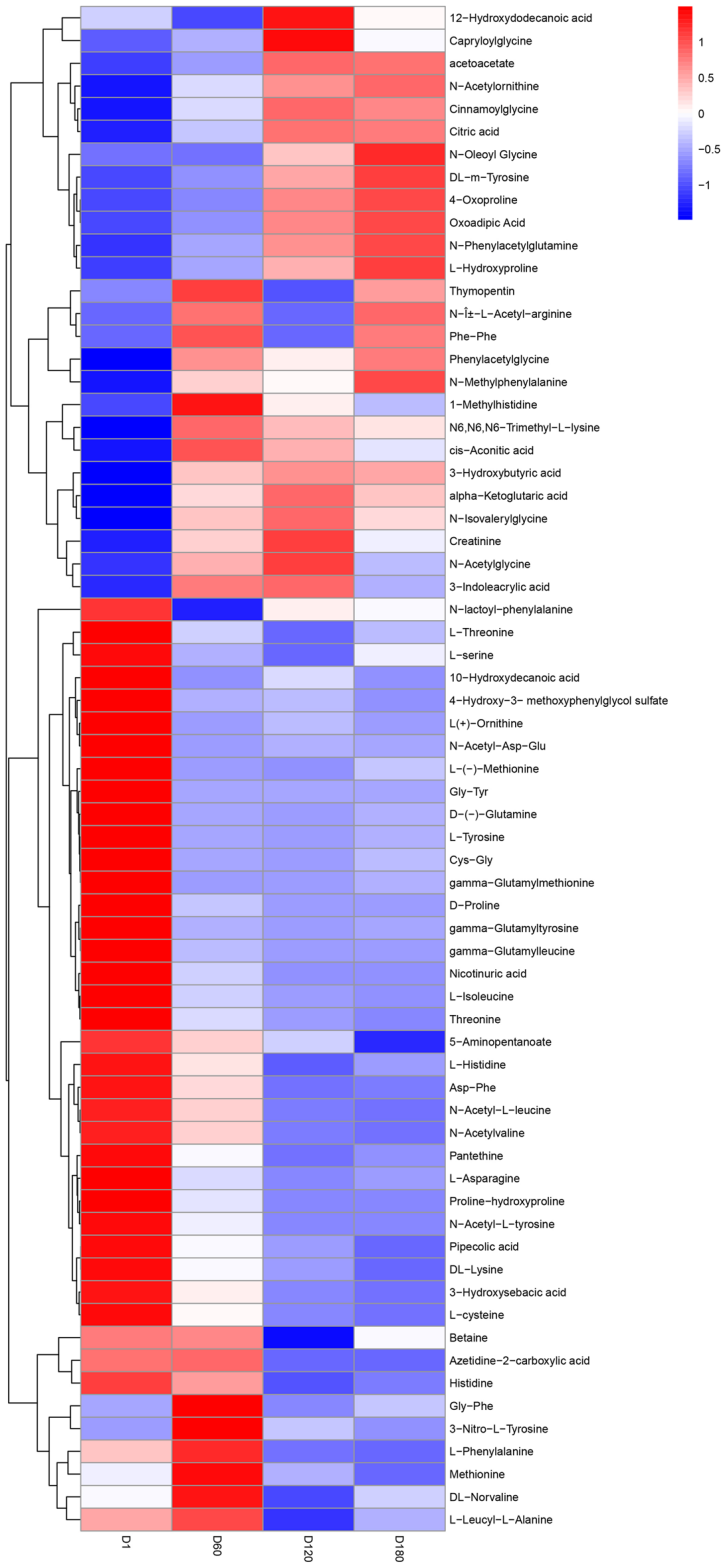
Heatmap of organic acid metabolites in the serum of Jining grey goats at different growth stages. Metabolites with high expression levels are represented by red squares, while those with low expression levels are indicated by blue squares.

A total of 63 organic acid metabolites were identified in our study (Fig. 6). These organic acids can be categorized into four expression patterns. Most amino acid-related metabolites such as l-tyrosine, dl-lysine, d-proline, gamma-glutamylleucine, gamma-glutamylmethionine, gamma-glutamyltyrosine, ( −)-methionine, L( +)-ornithine, l-asparagine, l-cysteine, l-histidine, l-serine, *N*-acetyl-Asp-Glu, *N*-acetylglycine, *N*-acetyl-l-leucine, nicotinuric acid, l-isoleucine, pantethine, proline-hydroxyproline, and threonine showed the highest levels after birth, which gradually decreased with age. Betaine, azetidine-2-carboxylic acid, l-phenylalanine, methionine, dl-norvaline, and l-leucyl-l-alanine exhibited the highest expression levels at D60 and then gradually declined. On the other hand, metabolites such as 12-hydroxydodecanoic acid, capryloylglycine, acetoacetate, *N*-acetylornithine, oxoadipic acid, and others demonstrated relatively low expression levels from D1 to D60, with an increasing trend with age. Thymopentin, *N*-methylphenylalanine, 1-methylhistidine, creatinine, *N*-acetylglycine, and 3-indoleacrylic acid, on the other hand, exhibited an increase in expression levels from D1 to D60, followed by a relatively stable pattern.

### Serum differential metabolite KEGG analysis

**Figure 7:**
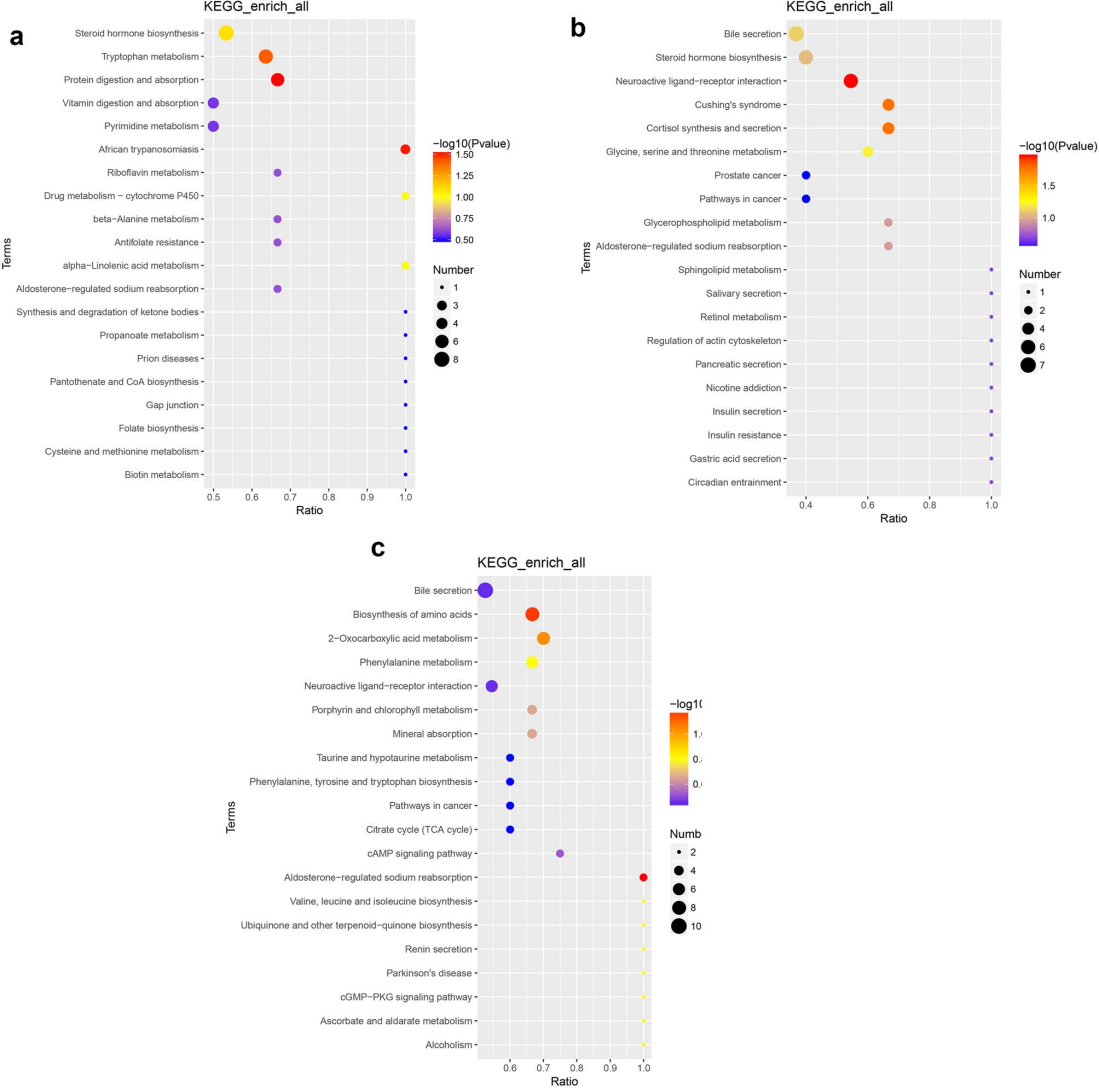
The top 20 KEGG pathways were identified from the age-associated differential accumulated metabolites (DAMs) in the serum metabolome of Jining Grey goats. (**a**) Comparison of D60 to D1. (**b**) Comparison of D120 to D60. (**c**) Comparison of D180 to D120.

To further explore the metabolic pathways related to age-related changes in goat growth and development, different metabolites were compared between D60 and D1, D120 and D60, and D180 and D120 groups, and KEGG analysis was performed on these differential metabolites. Figure 7 illustrates the top 20 enriched pathways in each comparison group. The results indicate that the different metabolites in the D60 vs. D1 group are enriched in pathways such as aldosterone-regulated sodium reabsorption, amino acid biosynthesis, 2-oxocarboxylic acid metabolism, and phenylalanine metabolism (Fig. 7a). In the D120 vs. D60 group (Fig. 7b), the different metabolites are enriched in pathways including protein digestion and absorption, African trypanosomiasis, tryptophan metabolism, and steroid hormone biosynthesis. As for the D180 vs. D120 group (Fig. 7c), the different metabolites are enriched in pathways such as neuroactive ligand-receptor interaction, cortisol synthesis and secretion, glycine, serine, and threonine metabolism, bile secretion, and steroid hormone biosynthesis. Tryptophan metabolism, protein digestion and absorption, and neuroactive ligand-receptor interaction are significantly enriched metabolic pathways throughout the age-related growth and development of goats (*P* < 0.05).

### WGCNA analysis

**Figure 8:**
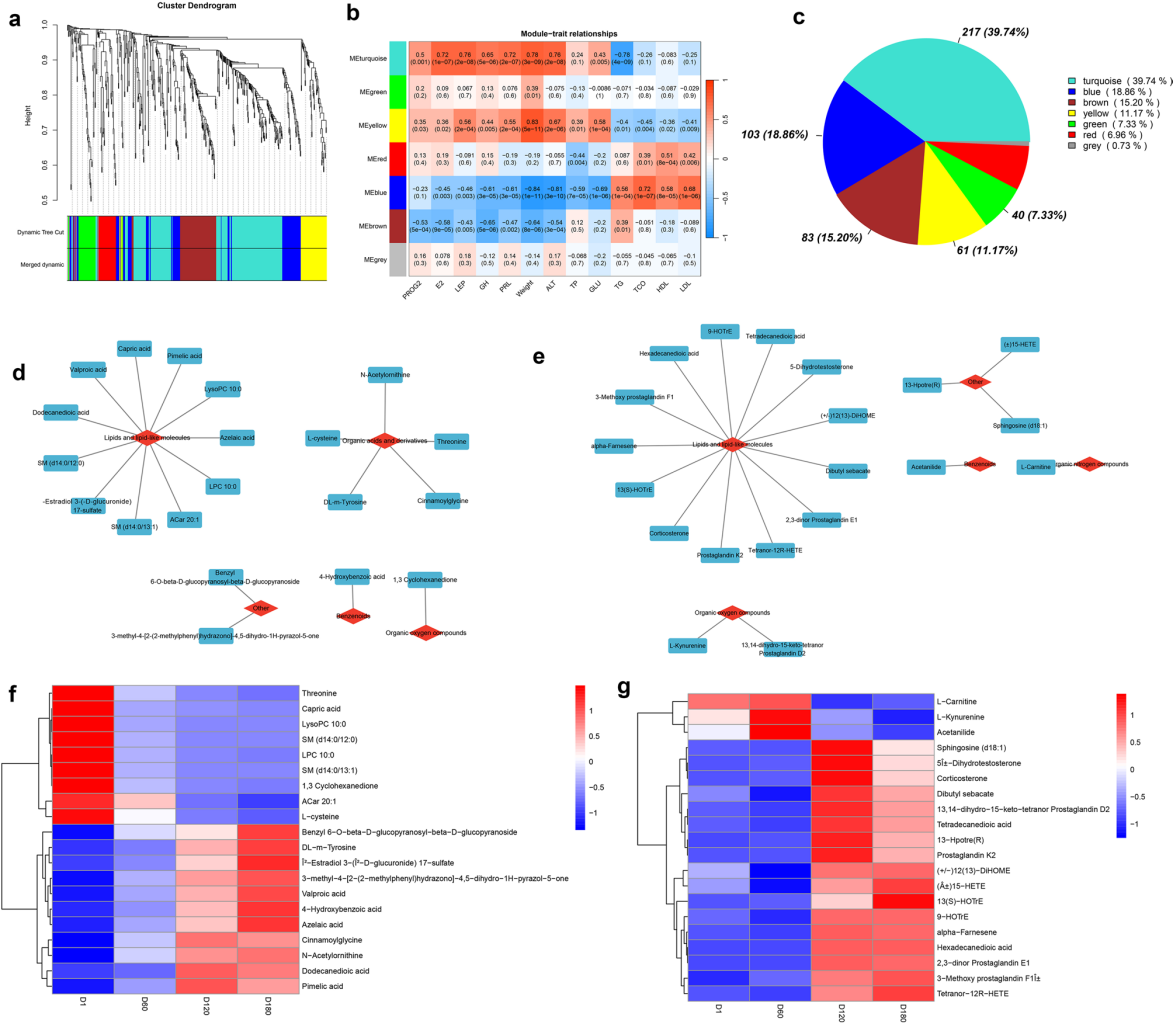
Correlation analysis of differential metabolites with phenotypic indicators such as body weight, serum hormones, and serum biochemistry in Jining Grey goats based on WGCNA analysis. (**a**) Hierarchical clustering tree of differential metabolites and module segmentation. (**b**) Correlation between modules and phenotypes. (**c**) Statistical analysis of the number of differential metabolites within each module. (**d**,**e**) Classification of hub metabolites in the Turquoise and Yellow modules. (**f**,**g**) Heatmap of hub metabolites in the Turquoise and Yellow modules.

To study the dynamic changes of serum metabolites during goat growth and development, the dynamic characteristics of metabolite accumulation related to serum biochemical indexes and hormone change levels were further analyzed by constructing WGCNA correlation networks (Fig. 8a). A total of seven co-expression modules were identified, and unselected metabolites were classified into grey modules. Based on the results of the correlation between modules and samples (Fig. 8b), we selected Turquoise, Yellow 2 modules, in which the Turquoise module contained 217 metabolites and showed significant positive correlation with body weight, P, E, GH, LEP, IGF-1, ALT indexes (r ≥ 0.5, *P* < 0.05), and most of the metabolites belonged to lipids and organic acids. According to |KME|> 0.5, the top 20 metabolites were identified as hub DAMs (Fig. 8c,d,f), where lipids and lipid-like molecules were predominant, and they were divided into two expression patterns.

Cluster analysis revealed that 1,3 cyclohexanedione, ACar 20:1, l-cysteine, and SM (d14:0/13:1) were highly expressed at 1 day of postnatal life and significantly decreased afterwards. On the other hand, Benzyl 6-*O*-beta-beta-glucopyranoside was also highly expressed at 1 day of postnatal life but declined remarkably. Moreover, -d-glucopyranosyl-beta-d-onine, capric acid, LysoPC 10:0, SM (d14:0/12:0), LPC 10:0, glucopyranoside, dl-m tyrosine, dodecanedioic acid and similar metabolites exhibited the lowest expression on the first day after birth, gradually increasing with age, and reaching their highest expression at 180 days.

The yellow module exhibited significant positive correlations with LEP, IGF-1, and weight traits (r ≥ 0.5, *P* < 0.05). This module comprised 67 metabolites, predominantly lipids. 20 hub metabolites showed two expression patterns (Fig. 8c,e,g): l-carnitine, l-kynurenine, and acetanilide were highly expressed in D60 while sphingosine (d18:1), 5β ± dihydrotestosterone, corticosterone, dibutyl sebacate, 13,14-dihydro-15-keto-tetranor prostaglandin D2, tetradecanedioic acid, 13-Hpotre(R), and 17 other metabolites were highly expressed during D120 and D180. These findings imply that lipids may play a crucial role in serotonin secretion and body weight gain during sexual maturation in goats.

## Discussion

Growth performance and sexual development are important traits in goats, and precocious puberty can shorten the era interval and improve animal performance. In recent years, metabolomics has gained wide application in livestock, through the identification of metabolites, it can realize the monitoring and assessment of livestock physiological state and nutritional level, etc., and characterize the differences in growth and milk production performance^20,21^. In this study, we first analyzed the differences in serum biochemical levels and serum hormone levels in goats of different days old. In addition, based on the non-targeted metabolomics technology of LC–MS/MS, the study analyzed the changes in serum metabolite levels in 1, 60, 120, and 180 days old goats, which is helpful to understand the changes in physiological state during goat development.

Serum biochemical indicators can directly reflect the nutritional level, metabolism and health status of the organism^22^. In this study, there were significant differences in the serum levels of ALT, TP, CK, GLU, TG, TC, HDL and LDL in the serum of Jining grey goats at different developmental stages (*P* < 0.05). ALT is predominantly found in animal livers and is often used as an indicator of stress and liver function^23–25^. Studies have shown that serum ALT levels in sheep vary with seasons and reproductive status^26^. The ALT levels in our study are consistent with Tschuor’s et al. study on goats^27^. The ALT level of Jining grey goats showed an upward trend after birth, which may be the result of the change of physiological state during the transition period from birth to sexual maturity. Total protein has an important impact on lamb immunity and growth^28^. Previous studies have shown that serum TP levels increase with sow age^29^. In our study, the TP content at 180 days of age was significantly higher than that of the other three stages, which may be due to the fact that the growth and development rate of goats increased with age, and their protein absorption and metabolic capacity gradually improved. GLU is the main source of energy for ruminants. Studies have shown that goats have low blood sugar levels after birth and gradually increase with age^30^. In this study, goats had slightly higher blood glucose levels than D60 after birth, possibly related to the goats’ postnatal colostrum intake to maintain energy needs^31,32^. With age, the increase in glucose levels may be due to increased glucose metabolism in the liver due to increased energy requirements for growth and sexual activity^33^. TG are the main form of fatty acids stored and transported inside cells and in blood plasma^34^. In humans and rodents, plasma TG metabolism changes with age^35^. The high triglyceride content of goats after birth may be related to the accelerated metabolism of fat to maintain basic life activities. TC refers to all lipoproteins in the body, which can participate in a variety of metabolic processes in liver cells and are raw materials for the synthesis of active substances such as steroid hormones, vitamin D, and bile acids^36^. There have been studies that have observed a rapid rise in plasma lipids in calves and goat calves in the weeks after birth^37,38^. LDL and HDL are important apolipoproteins that can bind to cholesterol in the serum^39^. The results showed that TG, LDL and HDL contents increased first and then decreased with the increase of age, and reached the highest value at 60 days of age, indicating that lipid metabolism of Jining grey goats was the most active at 60 days of age, which may be related to physiological adaptation during sexual maturation of goats. This results suggest that changes in age and physiological status affect various metabolic activities of goats, leading to changes in serum biochemistry.

In addition, this study examined the serum concentrations of hormones that are important regulators of growth and development in goats. The results showed that the levels of LEP, IGF-1, GH, P and E2 of Jining grey goats had significant changes from birth to 6 months of age, and all showed a trend of first increasing and then decreasing, reaching a peak at D120. Studies have shown that postnatal levels of sex hormones, follicle-stimulating hormone, and luteinizing hormone are lowest in sheep^40^, and as sexual development matures, LH and FSH secretion increases^41^, leading to an increase in gonadal steroid hormones^42,43^. This is consistent with our findings, which showed that the levels of estrogen and progesterone in Jining grey goat were the lowest after birth, and then the levels gradually increased. Continued elevation of reproductive hormone levels after puberty may be associated with follicular growth and ovulation^42^ until sexual maturity is reached when the mechanisms of estrogen-induced LH surge mature and regular ovulation cycles occur^44^. In addition, during puberty, elevated concentrations of sex steroids (especially estrogen) stimulate growth hormone production, activate the IGF-1 axis, and increase the levels of GH and IGF-1^45^. Leptin has a regulatory effect on GH secretion, and can also participate in the regulation of sexual maturation in animals by participating in energy homeostasis, regulating the secretion function of the HPG axis^46^. This is consistent with our findings, and the serum leptin and IGF-1 levels in Jining grey goats showed a similar pattern of change as reproductive hormones, suggesting that these hormones interact with each other during sexual maturation and jointly regulate the development and growth of goats.

In this study, a total of 1058 metabolites, including lipids and lipid-like molecules, organic acids and their derivatives, organic heterocyclic compounds, organic oxides, phenylcycles, nucleotides, phenylpropanoids and polyketides, and organic nitrogens, were identified in the serum samples from goats at four different developmental stages through comparative analysis of serum metabolomes. The results of PCA showed that the distance between D120 and D180 was closer, suggesting that the differences of the metabolites in the serum of goats mainly occurred between D1, D60 and D120, and the metabolite differences between D180 and D120 groups were smaller, which might be related to the similar physiological conditions of Jining grey goats in D120 and D180 groups. Further, a total of 504 different metabolites were identified, among which D60 vs. D1 had the largest number of differential metabolites, and the DAMs number gradually decreased with the increase of age.

Fatty acids and lipid compounds were detected as the most abundant type of differential metabolites in this study, mainly including glycerophospholipids, fatty acids, steroids and steroid derivatives, and sphingomyelin lipids. Lipids serve as structural componnents of membranes, energy storage and signalling molecules and help regulate many metabolic and immune processes. Glycerophospholipids are mainly composed of PE, PC, Lyso-PE and Lyso-PC, and phosphatidylcholine (PC) is the most abundant phospholipid in mammalian cell membranes, accounting for about 50% of the total phospholipid mass^47,48^. It has been shown that 40% of PC in blood LDL is converted to lyso-PC during oxidative modification^48^ and is highest in serum HDL^49^. In this study, elevated PC content may be associated with changes in serum HDL and LDL.FAHFAs belong to the fatty acid type of metabolites containing two fatty acids linked by hydroxy ester groups^50^, found in mammalian tissues and serum ^51^. Some FAHFAs have been shown to promote glucose metabolism, improve glucose homeostasis, and have some anti-inflammatory effects^52,53^. It is noteworthy that in our study, 13 specific FAHFA family members showed highly consistent differences among different day ages, with low levels of content after birth and a gradual increase in content with increasing age, especially from the D60 to D120 period. This may be due to the immature digestive system, weak metabolism and immunity of goats after birth and limited metabolism of FAHFAs. With increasing days, the growth and development of goats were gradually perfected, and the content of FAHFAs in serum was increased to satisfy the period energy and growth and development needs.

KEGG enrichment analysis showed that amino acid biosynthesis, phenylalanine metabolism, protein digestion and absorption, tryptophan metabolism, steroid hormone biosynthesis and neuroactive ligand receptor interaction were important pathways of serum metabolism in goats at different developmental stages. Indole-3-acetic acid, l-kynurenine, serotonin, indole, l-tryptophan, indole-3-acetaldehyde and *N*-formylkynurenine were metabolites enriched in tryptophan metabolism pathway. l-tryptophan is an essential amino acid that, in addition to being involved in protein synthesis and energy metabolism^54^, can also be used as a precursor to bioactive substances such as serotonin, melatonin, and kynurenine, thereby regulating physiological functions such as feeding behavior, reproduction, nutritional metabolism, and immunity in livestock^55,56^. In addition, dietary supplementation with Trp also increased cashmere goat body weight and improved sow reproductive performance and Serotonin and calcium concentrations^57,58^. Kynurenine is the main metabolite of Trp degradation^59^, has immunoprotective or neuroactive activity, and plays an important role in the early normal development of the nervous system in embryos and neonates^60^. Serotonin is a metabolite of tryptophan that is converted from tryptophan in the central nervous system (CNS), particularly in the hypothalamus. Serotonin acts as a neurotrophic factor in brain development, regulating neuronal development and synaptogenesis^61–63^. Induces GnRH and LH release in early prepubertal female rats and has inhibitory effects in adult female rats^64^. In addition, the serotonin system is involved in the development of the HPG axis and has a regulatory effect on ovarian sex hormone secretion^65^. In this study, tryptophan, kynurenine, and serotonin were all expressed at high levels of D60, and these DAMs may play an important role in goat gonadal axis reproductive activity and growth and development through tryptophan metabolism pathway.

WGCNA is a correlation-based method that describes and visualizes a network of data points and is now widely used in plant and animal metabolomics research^19,66–68^. Metabolites with similar expression patterns can be clustered, and co-expression networks can be constructed to analyze the relationship between phenotypes and metabolites and screen key metabolites, which can help reveal underlying biological mechanisms^69^. To further study the dynamic changes of serum metabolites during the growth and development of goats, some key metabolites, such as l-carnitine, threonine, and cysteine, were identified by WGCNA. l-carnitine promotes the β oxidation of long-chain fatty acids across mitochondrial membranes, which in turn promotes energy metabolism^70^. Dietary supplementation with l-carnitine has been shown to increase the growth rate of weaned calves and feed conversion in lambs^71,72^, and maternal l-carnitine supplementation has been shown to promote the development and thermogenesis of white adipose tissue in neonatal goats^73^. l-carnitine supplementation has been shown to increase plasma IGF-1 levels^74,75^ and play an important role in muscle cell proliferation^76^ and increase birth weight^77^. The results of this study showed that the higher content of l-carnitine from birth to D60 in Jining grey goats may be related to the promotion of thermogenesis by the white adipose tissue of newborn goats, and is involved in goat energy metabolism and protein deposition. Threonine is an important part of the body’s proteins, which can promote animal growth, enhance the body’s immunity, and maintain intestinal health^78,79^. Wellington et al. showed that the increased utilization of threonine during immune system stimulation in growing pigs may be related to threonine promoting the synthesis of many acute-phase proteins and immunoglobulins during the immune response^80,81^. In our study, threonine was highest at D1, which may be due to the high amount of immunoglobulins in goat colostrum, which is the most important amino acid that makes up IGG^82^. Newborn goats suck on colostrum, thus gaining passive immunity, preventing infection of goats by pathogens in the external environment. As we age, our immune system gradually improves, active immunity increases, and threonine levels decrease. Cysteine can be metabolized to produce taurine, glutathione, and hydrogen sulfide^83^, which has a variety of physiological functions such as antioxidant, reducing damage, and promoting growth^83–85^. In addition, l-cysteine has been shown to promote prepubertal goat oocyte maturation^86,87^. In this study, cysteine levels showed a decreasing trend after birth, which may be related to the growth and development of goats after birth, and may also be involved in oocyte maturation during sexual maturation. These results suggest that serum amino acid metabolism levels may play an important role in goat energy metabolism and sexual development.

## Conclusion

In summary, serum biochemical indexes and serum hormone contents undergo significant changes at different developmental stages of goats. The results indicated that PE, PC, Lyso-PE and Lyso-PC and FAFHA may play important roles in lipid metabolism in goats after birth. l-carnitine may be a metabolite related to muscle development in goats. Tryptophan metabolism, protein digestion and absorption and neuroactive ligand-receptor interactions were age-related metabolic pathways during growth and development of goats (*P* < 0.05). The results of the study depicted the dynamic changes of serum metabolites at different ages in Jining grey goats and supplemented the gap of serum metabolic changes at different ages after birth in goats.

### Supplementary Information

Supplementary Figures.

Supplementary Tables.

## Data Availability

Data is available in the article and supplementary information. The datasets generated and/or analysed during the current study are available in the Metabolights repository (MTBLS8944, https://www.ebi.ac.uk/metabolights/reviewer884ac141-297a-4968-bd8e-8fe093f36a26).

## Abbreviations

ALT: Alanine aminotransferase
AST: Aspartate aminotransferase
ALP: Alkaline phosphatase
TP: Total protein
ALB: Albumin
GLB: Globulin
CK: Creatine kinase
GLU: Glucose
TG: Triglycerides
TC: Total cholesterol
HDL: High-density lipoprotein
LDL: Low-density lipoprotein
GnRH: Gonadotropin-releasing hormone
FSH: Follicle stimulating hormone
LH: Luteinizing hormone
P: Progesterone
E: Estradiol

## References

[1] MacHugh DE, Bradley DG (2001). Livestock genetic origins: Goats buck the trend. Proc. Natl. Acad. Sci. USA.

[2] Miller BA, Lu CD (2019). Current status of global dairy goat production: An overview. Asian Australas. J. Anim. Sci..

[3] Sejian V (2021). Heat stress and goat welfare: Adaptation and production considerations. Anim. Open Access J. MDPI.

[4] Iommelli P, Infascelli L, Tudisco R, Capitanio F (2022). The Italian Cilentana goat breed: Productive performances and economic perspectives of goat farming in marginal areas. Trop. Anim. Health Prod..

[5] Fiehn O (2002). Metabolomics—The link between genotypes and phenotypes. Plant Mol. Biol..

[6] Oliver DJ, Nikolau B, Wurtele ES (2002). Functional genomics: High-throughput mRNA, protein, and metabolite analyses. Metab. Eng..

[7] Shulaev V (2006). Metabolomics technology and bioinformatics. Brief. Bioinform..

[8] Weckwerth W (2003). Metabolomics in systems biology. Ann. Rev. Plant Biol..

[9] Song B (2022). Comparisons of carcass traits, meat quality, and serum metabolome between Shaziling and Yorkshire pigs. Anim. Nutr..

[10] Zhang J, Gao Y, Guo H, Ding Y, Ren W (2019). Comparative metabolome analysis of serum changes in sheep under overgrazing or light grazing conditions. BMC Vet. Res..

[11] Wang DD (2023). Differences in serum metabolome profile explain individual variation in growth performance of young goats. J. Proteom..

[12] Batchu P, Terrill TH, Kouakou B, Estrada-Reyes ZM, Kannan G (2021). Plasma metabolomic profiles as affected by diet and stress in Spanish goats. Sci. Rep..

[13] Huang Y (2023). Effects of perinatal stress on the metabolites and lipids in plasma of dairy goats. Stress Biol..

[14] Shi Y, Wang S, Bai S, Huang L, Hou Y (2015). Postnatal ovarian development and its relationship with steroid hormone receptors in Jining Grey goats. Anim. Reprod. Sci..

[15] Wang Y (2023). Characterization of MicroRNA expression profiles in the ovarian tissue of goats during the sexual maturity period. J. Ovar. Res..

[16] Dai W (2017). Characterization of white tea metabolome: Comparison against green and black tea by a nontargeted metabolomics approach. Food Res. Int..

[17] Wen B, Mei Z, Zeng C, Liu S (2017). metaX: A flexible and comprehensive software for processing metabolomics data. BMC Bioinform..

[18] Kumar L, Futschik ME (2007). Mfuzz: A software package for soft clustering of microarray data. Bioinformation.

[19] Langfelder P, Horvath S (2008). WGCNA: An R package for weighted correlation network analysis. BMC Bioinform..

[20] Xue MY, Sun HZ, Wu XH, Liu JX, Guan LL (2020). Multi-omics reveals that the rumen microbiome and its metabolome together with the host metabolome contribute to individualized dairy cow performance. Microbiome.

[21] Metzler-Zebeli BU (2019). Feed restriction reveals distinct serum metabolome profiles in chickens divergent in feed efficiency traits. Metabolites.

[22] Sun JJ (2020). Effect of *Moringa oleifera *supplementation on productive performance, colostrum composition and serum biochemical indexes of sow. J. Anim. Physiol. Anim. Nutr..

[23] Senior JR (2012). Alanine aminotransferase: A clinical and regulatory tool for detecting liver injury-past, present, and future. Clin. Pharmacol. Ther..

[24] Piccione G (2010). Reference values for some haematological, haematochemical, and electrophoretic parameters in the Girgentana goat. Turk. J. Vet. Anim. Sci..

[25] Stojević Z, Pirsljin J, Milinković-Tur S, Zdelar-Tuk M, Beer Ljubic B (2005). Activities of AST, ALT and GGT in clinically healthy dairy cows during lactation and in the dry period. Vet. Arh..

[26] Yokus B, Cakir DU, Kanay Z, Gulten T, Uysal E (2006). Effects of seasonal and physiological variations on the serum chemistry, vitamins and thyroid hormone concentrations in sheep. J. Vet. Med. Physiol. Pathol. Clin. Med..

[27] Tschuor AC, Riond B, Braun U, Lutz HJSAFT (2008). Hmatologische und klinisch-chemische referenzwerte fr adulte ziegen und schafe. Schweiz. Arch. Fur Tierheilkd..

[28] Chen JC, Chang CJ, Peh HC, Chen SYJSRR (1999). Serum protein levels and neonatal growth rate of Nubian goat kids in Taiwan area. Small Rumin. Res..

[29] Friendship RM, Lumsden JH, McMillan I, Wilson MR (1984). Hematology and biochemistry reference values for Ontario swine. Can. J. Comp. Med. Rev. Can. Med. Comp..

[30] Habibu B, Umaru Kawu M, Aluwong T, Joan Makun H (2021). Postnatal hypoglycemia and blood glucose concentrations in neonatal tropical goat kids. Vet. Clin. Pathol..

[31] Abdolvahabi S, Zaeemi M, Mohri M, Naserian A (2016). Age related changes in serum biochemical profile of Saanen goat kids during the first three months of life. Rev. Méd. Vét..

[32] Madan J, Sindhu S, Rose MK (2020). Changes in plasma biochemical parameters and hormones during transition period in Beetal goats carrying single and twin fetus. Vet. World.

[33] De Koster JD, Opsomer G (2013). Insulin resistance in dairy cows. Vet. Clin. N. Am. Food Anim. Pract..

[34] Chen Q (2018). Chronic dexamethasone exposure markedly decreased the hepatic triglyceride accumulation in growing goats. Gen. Comp. Endocrinol..

[35] Spitler KM, Davies BSJ (2020). Aging and plasma triglyceride metabolism. J. Lipid Res..

[36] Nemes K, Åberg F, Gylling H, Isoniemi H (2016). Cholesterol metabolism in cholestatic liver disease and liver transplantation: From molecular mechanisms to clinical implications. World J. Hepatol..

[37] Jenkins KJ, Griffith G, Kramer JK (1988). Plasma lipoproteins in neonatal, preruminant, and weaned calf. J. Dairy Sci..

[38] Bogin E, Shimshony A, Avidar Y, Israeli B (1981). Enzymes, metabolites and electrolytes levels in the blood of local Israeli goats. Zent. Fur Vet. R. A.

[39] Li X, Du Y, Zhang C, Tu Z, Wang L (2022). Modified highland barley regulates lipid metabolism, liver inflammation and gut microbiota in high-fat/cholesterol diet mice as revealed by LC–MS based metabonomics. Food Funct..

[40] Georgieva RI, Bulahbel S, Georgiev HG (1994). Patterns of variations in FSH, LH and 17beta-estradiol during the postnatal development of sheep. Theriogenology.

[41] Chellakooty M (2003). Inhibin A, inhibin B, follicle-stimulating hormone, luteinizing hormone, estradiol, and sex hormone-binding globulin levels in 473 healthy infant girls. J. Clin. Endocrinol. Metab..

[42] Messinis IE (2006). From menarche to regular menstruation: Endocrinological background. Ann. N. Y. Acad. Sci..

[43] Howard SR (2021). Interpretation of reproductive hormones before, during and after the pubertal transition-identifying health and disordered puberty. Clin. Endocrinol..

[44] DiVall SA, Radovick S (2008). Pubertal development and menarche. Ann. N. Y. Acad. Sci..

[45] Mauras N, Rogol AD, Haymond MW, Veldhuis JD (1996). Sex steroids, growth hormone, insulin-like growth factor-1: Neuroendocrine and metabolic regulation in puberty. Horm. Res..

[46] Martos-Moreno GA, Chowen JA, Argente J (2010). Metabolic signals in human puberty: Effects of over and undernutrition. Mol. Cell. Endocrinol..

[47] Vance JE, Vance DE (2004). Phospholipid biosynthesis in mammalian cells. Biochem. Cell Biol. Biochim. Et Biol. Cell..

[48] Kent C (1995). Eukaryotic phospholipid biosynthesis. Ann. Rev. Biochem..

[49] Dashti M (2011). A phospholipidomic analysis of all defined human plasma lipoproteins. Sci. Rep..

[50] Brejchova K (2020). Understanding FAHFAs: From structure to metabolic regulation. Prog. Lipid Res..

[51] Yore MM (2014). Discovery of a class of endogenous mammalian lipids with anti-diabetic and anti-inflammatory effects. Cell.

[52] Balas L, Feillet-Coudray C, Durand T (2018). Branched fatty acyl esters of hydroxyl fatty acids (FAHFAs), appealing beneficial endogenous fat against obesity and type-2 diabetes. Chemistry.

[53] Lee J (2016). branched fatty acid esters of hydroxy fatty acids (FAHFAs) protect against colitis by regulating gut innate and adaptive immune responses. J. Biol. Chem..

[54] Priatno W (2020). “Dietary supplementation of L-tryptophan” increases muscle development, adipose tissue catabolism and fatty acid transportation in the muscles of Hanwoo steers. J. Anim. Sci. Technol..

[55] Moehn S, Pencharz PB, Ball RO (2012). Lessons learned regarding symptoms of tryptophan deficiency and excess from animal requirement studies. J. Nutr..

[56] Yao K (2011). Tryptophan metabolism in animals: Important roles in nutrition and health. Front. Biosci..

[57] Ma H, Zhang W, Song WH, Sun P, Jia ZH (2012). Effects of tryptophan supplementation on cashmere fiber characteristics, serum tryptophan, and related hormone concentrations in cashmere goats. Domest. Anim. Endocrinol..

[58] Miao J (2019). Tryptophan supplementation increases reproduction performance, milk yield, and milk composition in lactating sows and growth performance of their piglets. J. Agric. Food Chem..

[59] Platten M, Nollen EAA, Röhrig UF, Fallarino F, Opitz CA (2019). Tryptophan metabolism as a common therapeutic target in cancer, neurodegeneration and beyond. Nature Rev. Drug Discov..

[60] Forrest CM, Khalil OS, Pisar M, Darlington LG, Stone TW (2013). Prenatal inhibition of the tryptophan-kynurenine pathway alters synaptic plasticity and protein expression in the rat hippocampus. Brain Res..

[61] Azmitia EC (2001). Modern views on an ancient chemical: Serotonin effects on cell proliferation, maturation, and apoptosis. Brain Res. Bull..

[62] Gaspar P, Cases O, Maroteaux L (2003). The developmental role of serotonin: News from mouse molecular genetics. Nature Rev. Neurosci..

[63] Deneris E, Gaspar P (2018). Serotonin neuron development: Shaping molecular and structural identities. WIREs. Dev. Biol..

[64] Moguilevsky JA, Wuttke W (2001). Changes in the control of gonadotrophin secretion by neurotransmitters during sexual development in rats. Exp. Clin. Endocrinol. Diabetes Off. J. Ger. Soc. Endocrinol. Ger. Diabetes Assoc..

[65] Koppan M, Bodis J, Verzar Z, Tinneberg HR, Torok A (2004). Serotonin may alter the pattern of gonadotropin-induced progesterone release of human granulosa cells in superfusion system. Endocrine.

[66] Murga-Garrido SM (2021). Gut microbiome variation modulates the effects of dietary fiber on host metabolism. Microbiome.

[67] Zhang C (2023). Multi-omics reveals that the host-microbiome metabolism crosstalk of differential rumen bacterial enterotypes can regulate the milk protein synthesis of dairy cows. J. Anim. Sci. Biotechnol..

[68] Li L (2023). Characterization of novel loci controlling seed oil content in Brassica napus by marker metabolite-based multi-omics analysis. Genome Biol..

[69] Pei G, Chen L, Zhang W (2017). WGCNA application to proteomic and metabolomic data analysis. Methods Enzymol..

[70] Pekala J (2011). l-carnitine–metabolic functions and meaning in humans life. Curr. Drug Metab..

[71] Solhjoo A, Eb R, Bayat A, Zamiri MJ (2014). The effect of rumen protected l-carnitine on feedlot performance, carcass characteristics and blood metabolites in Iranian fat-tailed Ghezel lambs. Res. Opin. Anim. Vet. Sci..

[72] Meyer J (2020). Effects of a dietary l-carnitine supplementation on performance, energy metabolism and recovery from calving in dairy cows. Anim. Open Access J. MDPI.

[73] Liu X (2022). Maternal l-carnitine supplementation promotes brown adipose tissue thermogenesis of newborn goats after cold exposure. FASEB J. Off. Publ. Fed. Am. Soc. Exp. Biol..

[74] Kita K, Kato S, Amanyaman M, Okumura J, Yokota H (2002). Dietary l-carnitine increases plasma insulin-like growth factor-I concentration in chicks fed a diet with adequate dietary protein level. Br. Poult. Sci..

[75] Brown KR (2008). Effects of feeding l-carnitine to gilts through day 70 of gestation on litter traits and the expression of insulin-like growth factor system components and L-carnitine concentration in foetal tissues. J. Anim. Physiol. Anim. Nutr..

[76] Florini JR (1991). “Spontaneous” differentiation of skeletal myoblasts is dependent upon autocrine secretion of insulin-like growth factor-II. J Biol Chem..

[77] Hills FA, English J, Chard T (1996). Circulating levels of IGF-I and IGF-binding protein-1 throughout pregnancy: Relation to birthweight and maternal weight. J. Endocrinol..

[78] Wang W, Zeng X, Mao X, Wu G, Qiao S (2010). Optimal dietary true ileal digestible threonine for supporting the mucosal barrier in small intestine of weanling pigs. J. Nutr..

[79] Chen Y (2018). Dietary l-threonine supplementation attenuates lipopolysaccharide-induced inflammatory responses and intestinal barrier damage of broiler chickens at an early age. Br. J. Nutr.

[80] Wellington MO, Htoo JK, Van Kessel AG, Columbus DA (2018). Impact of dietary fiber and immune system stimulation on threonine requirement for protein deposition in growing pigs. J. Anim. Sci..

[81] Li P, Yin YL, Li D, Kim SW, Wu G (2007). Amino acids and immune function. Br. J. Nutr..

[82] Tenenhouse HS, Deutsch HF (1966). Some physical-chemical properties of chicken gamma-globulins and their pepsin and papain digestion products. Immunochemistry.

[83] Takagi H, Ohtsu I (2017). l-cysteine metabolism and fermentation in microorganisms. Adv. Biochem. Eng. Biotechnol..

[84] Iqbal S (2016). l-Cysteine improves antioxidant enzyme activity, post-thaw quality and fertility of Nili-Ravi buffalo (Bubalus bubalis) bull spermatozoa. Andrologia.

[85] Bilodeau JF, Chatterjee S, Sirard MA, Gagnon C (2000). Levels of antioxidant defenses are decreased in bovine spermatozoa after a cycle of freezing and thawing. Mol. Reprod. Dev..

[86] Rodríguez-González E, López-Bejar M, Mertens MJ, Paramio MT (2003). Effects on in vitro embryo development and intracellular glutathione content of the presence of thiol compounds during maturation of prepubertal goat oocytes. Mol. Reprod. Dev..

[87] Hong S, Vettical BS, Wani NA (2020). Development of prepubertal goat oocytes after their in vitro maturation and chemical activation. Zygote.

