# Supplementary material for: Comparative metabolomics reveals serum metabolites changes in goats during different developmental stages
Source: Sci Rep. 2024 Mar 27;14:7291. doi: 10.1038/s41598-024-57803-7 (PMC10973421; doi:10.1038/s41598-024-57803-7)
Supplement: Supplementary file 1 — Supplementary Figures. [file 41598_2024_57803_MOESM1_ESM.pdf]

Comparative metabolomics reveals serum metabolites changes in goats during different developmental stages

Qing Li [a, b]; Jianmin Wang [a, b]; Yanyan Wang [a, b]; Rong Xuan [a, b]; Yanfei Guo [a, b]; Peipei He [a, b;] Lu Zhang [a, b]; Tianle Chao [a, b]

a Shandong Provincial Key Laboratory of Animal Biotechnology and Disease Control and Prevention, College of Animal Science and Veterinary Medicine, Shandong Agricultural University, 271018 Tai'an City, Shandong Province, China.

[b] Key Laboratory of Efficient Utilization of Non-grain Feed Resources (Co-construction by Ministry and Province), Ministry of Agriculture and Rural Affairs, Shandong Agricultural University, 271018, Tai'an City, Shandong Province, China.

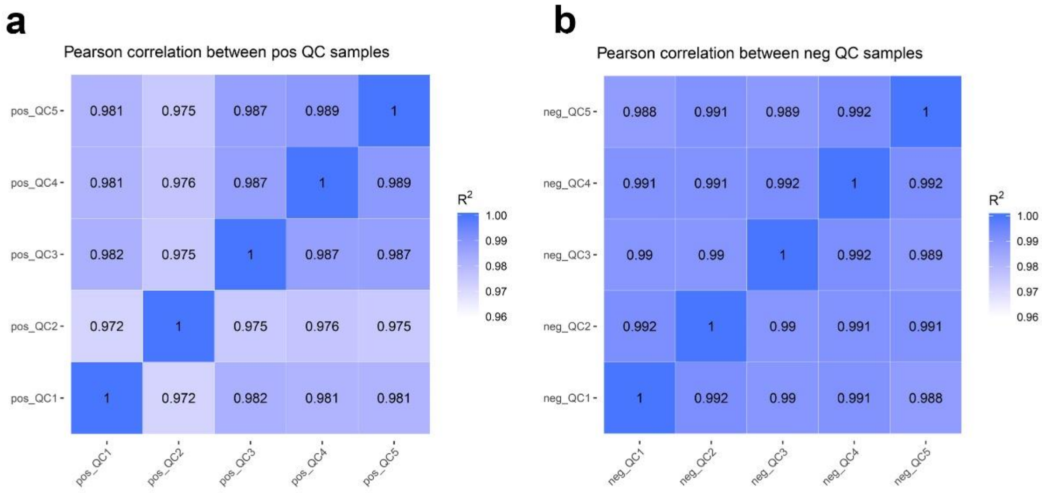

Figure S1. Quality control of serum metabolomics QC samples. (a). Heatmap of Pearson correlation between pos QC sample. (b). Heatmap of Pearson correlation between neg QC sample.

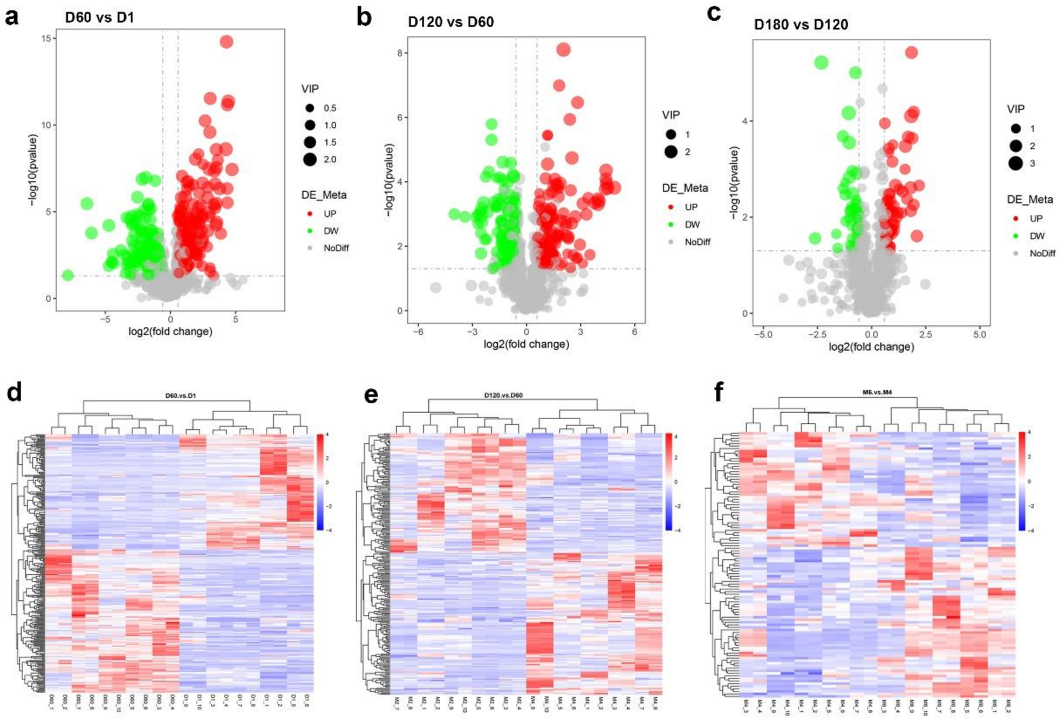

Figure S2. Overview of age-related differential metabolites in serum of Jining Grey goats at different growth stages. (a)-(c) Volcano plots of differential metabolites between D60vsD1, D120vsD60, D180vsD120. Differential metabolites (DAMs) were defined based on the following selection criteria: VIP value > 1, p-value (from t-test) < 0.05. (d)-(f) Heatmap of DAMs clustering between D60vsD1, D120vsD60, and D180vsD120

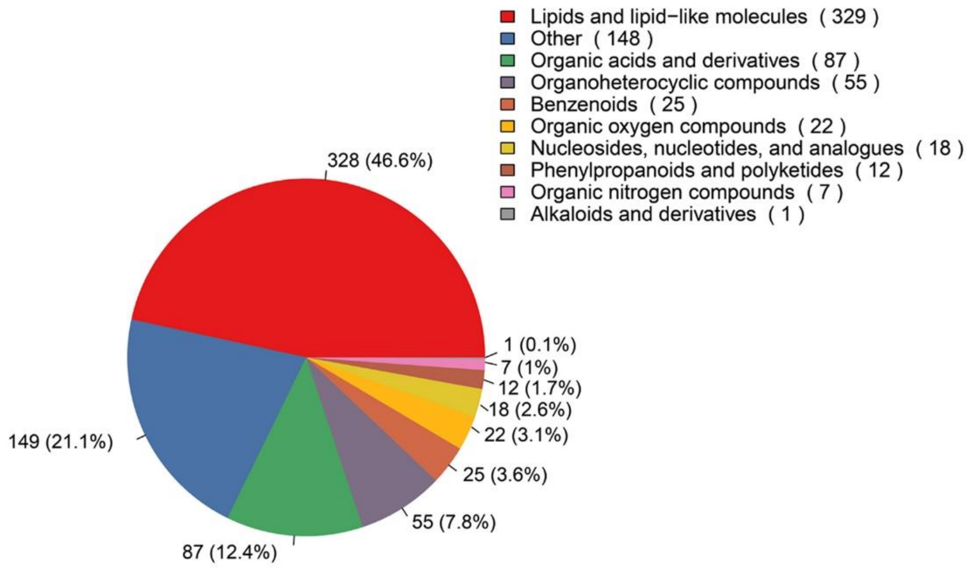

Figure S3 Composition analysis of differential metabolites in serum metabolomic profiles of Jining Grey goat at different ages.
